# Increasing the Number of Adverse Drug Reactions Reporting: the Role of Clinical Pharmacy Residents

**Published:** 2014

**Authors:** Shadi Baniasadi, Maryam Habibi, Roodabeh Haghgoo, Masoumeh Karimi Gamishan, Fatemeh Dabaghzadeh, Maryam Farasatinasab, Shadi Farsaei, Afshin Gharekhani, Hamidreza Kafi, Iman Karimzadeh, Ali Kharazmkia, Farhad Najmeddin, Naemeh Nikvarz, Mohammad Bagher Oghazian, Haleh Rezaee, Kourosh Sadeghi, Ali Tafazzoli, Nahid Shahsavari, Fanak Fahimi

**Affiliations:** a Virology Research Center, NRITLD, Masih Daneshvari Hospital, Shahid Beheshti University of Medical Sciences, Tehran, Iran.; b Chronic Respiratory Disease Research Center, NRITLD, Masih Daneshvari Hospital, Shahid Beheshti University of Medical Sciences, Tehran, Iran.; c Importing and Exporting Office, Food and Drug Organization, Ministry of Health and Medical Education, Tehran, Iran.; d Faculty of Pharmacy and Pharmaceutical Sciences, Kerman Medical University, Kerman, Iran.; e Clinical Pharmacy Department, School of Pharmacy, Shahid Beheshti University of Medical Sciences, Tehran, Iran.; f Department of Clinical Pharmacy and Pharmacy Practice, School of Pharmacy and Pharmaceutical Sciences, Isfahan University of Medical Sciences, Isfahan, Iran.; g Clinical Pharmacy Department, School of Pharmacy, Tehran University of Medical Sciences, Tehran, Iran.; h Lorestan University of Medical Sciences, Khorramabad, Iran.; i Infectious Diseases and Tropical Medicine Research Center, Department of Clinical Pharmacy, Faculty of Pharmacy, Tabriz University of Medical Sciences, Tabriz, Iran.

**Keywords:** Adverse drug reaction, Clinical pharmacist, Pharmacy resident, Hospital, Pharmacovigilance, Spontaneous reporting

## Abstract

Detection of adverse drug reactions (ADRs) in hospitals provides an important measure of the burden of drug related morbidity on the healthcare system. Spontaneous reporting of ADRs is scare and several obstacles to such reporting have been identified formerly. This study aimed to determine the role of clinical pharmacy residents in ADR reporting within a hospital setting. Clinical pharmacy residents were trained to report all suspected ADRs through ADR-reporting yellow cards. The incidence, pattern, seriousness, and preventability of the reported ADRs were analyzed. During the period of 12 months, for 8559 patients, 202 ADR reports were received. The most frequently reported reactions were due to anti-infective agents (38.38%). Rifampin accounted for the highest number of the reported ADRs among anti-infective agents. The gastro-intestinal system was the most frequently affected system (21.56%) of all reactions. Fifty four of the ADRs were reported as serious reactions. Eighteen of the ADRs were classified as preventable. Clinical pharmacy residents' involvement in the ADR reporting program could improve the ADR reporting system.

## Introduction

Adverse drug reactions (ADRs) are common causes of morbidity and mortality within the hospital setting. The hospital setting, with its clearly defined patient population, is an ideal setting to identify potential adverse drug reaction signals and to report them to either the drug company or the FDA(1) Reporting of ADRs is an important component of monitoring and evaluation activities performed in the hospitals. A productive hospital-based reporting program can be helpful in providing valuable information regarding potential problems of drug usage in an institution. Through these efforts, problems are identified and resolved, which results in continuous improvement in patient care (2).

Spontaneous reporting program, a common method of drug surveillance is capable of recognizing ADRs in the daily medical practice, even though underreporting and absence of information on ADRs are its disadvantages (3,4). Although identification and documentation of ADRs are considered as all health care professionals responsibility, pharmacists and especially clinical pharmacists are often in charge for these tasks (5).

Clinical pharmacy is a relatively a new concept in Iran (6). This new breed of pharmacy practices is more patient oriented rather than drug product oriented (7). Clinical pharmacy residents are in an ideal position to increase ADR reporting rates within health systems (8). ADR review can be added to the daily responsibilities of residents participating in the hospitals and clinical rotations. In this article we tried to demonstrate the contribution of clinical pharmacy residents in the process of ADR reporting within a hospital setting and assess the role of the clinical pharmacy residents on the number and reporting rate of ADRs.

## Exprimental

The study was conducted in Masih Daneshvari Hospital over a one year period from March 2010 to February 2011. During the orientation session at the beginning of each new rotation, residents received a brief overview of the hospital’s ADR reporting program. Residents began prospectively and retrospectively collecting data on suspected ADRs. Prospective ADRs were identified by physicians, nurses, or residents during interdisciplinary rounds. Retrospective ADRs were identified by reviewing patient’s records. The residents documented the suspected drug(s) for the event on yellow cards. Pharmaceutical care department staffs reviewed each ADR report and were responsible for the accuracy of the reported ADR. 

An ADR was defined according to the World Health Organization (WHO) definition as: “a response to a drug which is noxious and unintended, and which occurs at doses normally used in man for the prophylaxis, diagnosis, or therapy of disease, or for the modification of physiological function.”

Each ADR was classified according to the WHO system organ classification (9). All reported ADRs were evaluated for the causality in accordance with Naranjo’s algorithm (10). The seriousness of reported adverse reactions was assessed based on the WHO definition, which included any adverse event that resulted in death, life-threatening situation, hospitalization, prolonged hospital stay, disability or birth defect. Assessment of preventability was determined using the scale developed by Schumock *et al*. (11).

All documented ADRs were electronically entered into an Excel spreadsheet for the purpose of generating annually reports. ADR forms were then sent to the national ADR monitoring centre at the Ministry of Health for further evaluation.

The number and reporting rate (number of reports/number of beds) of ADRs were compared with our previous study (12). 

## Results

During 12 months, a total of 8559 patients were admitted to the hospital. Sixteen clinical pharmacy residents were rotating who reported 202 ADRs from 178 patients. A total of 202 ADRs were documented in this study, compared with 112 in previous study (12).

In terms of the patient demographics in the reported ADRs, 111 were women and 91 were men. 

Number of patients in pediatric (0–18 years), adult (19–60 years) and geriatric (> 60 years) groups were 11, 112 and 79, respectively. ADRs were reported from 8 wards. The majority of the ADR reports were from the Department of Internal Medicine (31.68%), followed by the Coronary Care Unit (16.83%) as presented in Table 1. The most frequent reports were due to anti-infective agents (38.38%), followed by antineoplastic agents (11.45%) and central nervous system agents (10.44%). The drug class involved in the ADRs is shown in Table 2.

**Table 1:** Wards associated with the reported ADRs

**Ward**	**Number of ADRs**	**Percentage**
Internal medicine	64	31.68
Cardiac Care Unit	34	16.83
Intensive Care Unit	29	14.36
Tuberculosis	28	13.86
Oncology	26	12.87
Surgery	9	4.46
Pediatric	7	3.47
Transplant	5	2.48

Rifampin was the most frequent anti-infective agent associated with the suspected ADRs such as nausea, vomiting, abdominal pain, urticaria, hepatic enzyme increased, pancytopenia, and Stevens-Johnson syndrome (SJS) (Table 3). 

**Table 2:** Drug class implicated in the reported ADRs

**Drug class**	**Number of ADRs**	**Percentage**
Anti-infective agents	114	38.38
Antineoplastic agents	34	11.45
Central nervous system agents	31	10.44
Cardiovascular drugs	24	8.08
Miscellaneous Therapeutic Agents	19	6.40
Blood formation and coagulation	17	5.72
Autonomic drugs	16	5.39
Hormones and synthetic substitutes	15	5.05
Electrolytic, Caloric, and Water Balance	11	3.70
Gastrointestinal drugs	9	3.03
Antitussives, Expectorants, and Mucolytic Agents	4	1.35
Antihistamine drugs	1	0.34
Vitamins	1	0.34
Eye, Ear, Nose, and Throat preparations	1	0.34

The gastrointestinal system was the most frequently affected system (21.56% of all reactions), followed by the skin and appendages system (14.50%). The classification of the ADRs by system-organ class is demonstrated in Table 4. The causality assessment of ADRs revealed that 35.64% as probable and 64.36% as possible reactions. Fifty four (26.73%) of the ADRs were classified as serious according to the WHO definition and resulted in prolonged hospital stay, persistent disability or death. Table 5 shows the drugs with a serious reaction. 

Eighteen (8.91%) of the ADRs were classified as preventable according to the scale developed by Schumock *et al. *(11).

**Table 3:** Anti-infective agents implicated in ADRs

**Anti-infective agents**	**Number of ADRs**	**Percentage**
Rifampin	23	20.35
Isoniazid	21	18.58
Pyrazinamide	18	15.93
Vancomycin	14	12.39
Clindamycin	5	4.42
Ceftriaxone	4	3.54
Co-trimoxazole	4	3.54
Clarithromycin	4	3.54
Ofloxacin	4	3.54
Ethambutol	3	2.65
Azithromycin	2	1.77
Dapsone	1	0.88
Erythromycin	1	0.88
Oseltamivir	1	0.88
Ganciclovir	1	0.88
Chloramphenicol	1	0.88
Linezolid	1	0.88
Nitrofurantoin	1	0.88
Penicillin	1	0.88
Cefalexin	1	0.88
Meropenem	1	0.88
Piperacillin	1	0.88

**Table 4:** Organ systems associated with the reported ADRs

**System associated with ADRs**	**Number of ADRs**	**Percentage**
Gastro-intestinal system	58	21.56
Skin and appendages	39	14.50
Central & peripheral nervous system	31	11.52
Metabolic and nutritional	22	8.18
Liver and biliary system	20	7.43
Platelet, bleeding & clotting	18	6.69
Respiratory system	13	4.83
Vascular (extracardiac)	12	4.46
Body as a whole-general	10	3.72
Psychiatric	10	3.72
Vision	6	2.23
Red blood cell	5	1.86
Urinary system	4	1.49
White cell	4	1.49
Musculo- skeletal system	4	1.49
Cardiovascular	3	1.12
Resistance mechanism	3	1.12
Heart rate and rhythm	2	0.74
Application site	2	0.74
Special senses other	2	0.74
Hearing and vestibular	1	0.37

**Table 5:** Drugs reported in serious ADRs by frequency of times reported and type of reaction

**Drugs**	**Type of reaction**	**Number of reports**
**Rifampin**	Hepatic enzyme increased	9
	Pancytopenia	1
	Stevens Johnson Syndrome	1
Isoniazid	Hepatic enzyme increased	8
Heparin	Thrombocytopenia	3
	Haematuria, Gastric ulcer hemorrhagic, Ecchymosis	1
Cyclosporine	Nephropathy toxic, Hepatic enzyme increased	2
	Vomiting, Nausea	1
Vancomycin	Renal failure	1
	Thrombocytopenia	1
Carboplatin	Pancytopenia	1
	Leucopenia, Anemia	1
Anti-thymocyte globulin	Thrombocytopenia	2
Insulin Regular	Hypoglycemia	2
Mycophenolate mofetil	Leucopenia, Neutropenia	2
Ranitidine	Thrombocytopenia	1
Allopurinol	Stevens Johnson Syndrome	1
Amiodarone	Thrombocytopenia, Anemia	1
Betaxolol	Dyspnoea, Bronchospasm	1
Carbamazepine	Dyspnoea, Dermatitis	1
Co-trimoxazole	Anemia	1
Digoxin	Thrombocytopenia	1
Cyclophosphamide	Pulmonary edema, Oedema peripheral	1
Enoxaparin	Thrombocytopenia	1
Hyoscine	Dyspnoea	1
Insulin NPH	Hypoglycemia	1
Morphine sulfate	Convulsion	1
Propranolol	Bronchospasm, Dyspnoea, Consciousness decreased	1
Pyrazinamide	BUN increased	1
Sodium valproate	Thrombocytopenia	1
Warfarin	Pulmonary haemorrhage	1
Ceftriaxone	Allergic reaction	1

## Discussion

The contribution of clinical pharmacy residents in improving ADR reporting was determined by comparing current with previous ADR data. A total of 202 ADRs were documented in this study, compared with 112 in previous study (12) an increase of almost twofold. Reporting rate of ADRs in our center was increased from 40% in year 2006 to 80% in year 2010.

Sullivan et al. showed that pharmacy student participation in the ADR reporting significantly increases the number of ADRs documented (5). 

Until recent years, colleges of pharmacy in Iran had no clinical education (13). Significant progress has been made since 1996 toward changing the emphasis of pharmaceutical education from preparing a product-oriented pharmacist to preparing a patient- oriented individual with good basic knowledge of drugs. These pharmacists can work in close association with physicians and others on the health care teams (14). It is the pharmacist’s responsibility and professional commitment to report any suspected ADRs (15).

In our study, the most frequent ADRs were related to antibiotics that are costy and highly consumed in our hospital. While in other studies, different drug classes such as antineoplastic, cardiovascular, anticoagulant, nonsteroidal anti-inflammatory, hypoglycemic, and anti-infective agents have been most frequently associated with ADRs (16-21).

Our previous study has shown ceftriaxone as the most frequent anti-infective agent associated with the suspected ADRs (12). Perhaps emphasis on the appropriate and rational use of this drug by pharmaceutical care department in the rounds, consultations, random medication reviews, or via newsletter, (22-25), has changed drug utilization pattern and consequently ADR pattern. 

In the present study rifampin has been replaced as the most frequent anti-infective agent associated with the suspected ADRs. It could be rational since our hospital is a tuberculosis referral center and therefore rifampin is highly prescribed. 

Gastro-intestinal system was the most reported organ system which was affected by ADRs. Skin and appendages systems have usually been reported as the most affected organ system by ADRs (26-28). This reveals that clinical pharmacy residents have used their clinical judgments which could be superior to documenting only those visible ADRs. Pathological and laboratory data which are objective markers of ADRs should be considered by residents (12).

In this study, 8.91% of ADRs were preventable. Higher rate of preventable ADRs in other studies (the wide range of 30-70%) (28, 29). and 13.39% reduction in preventable ADRs in our hospital (22.30% preventable ADRs in previous study) suggest that preventive strategies have been effective in our institute.

Serious ADRs have been increased from 16.70% (in previous study) to 26.73% (in this study). Perhaps clinical pharmacy residents are eligible to detect serious ADRs more efficiently. The assessment of whether an ADR has increased the length of stay or caused persistent disability or death can be difficult to assess because individual patient factors such as the nature and severity of the underlying disease, and social factors may also contributed to the length of stay (30) medication errors (31) and inappropriate prescribing patterns (32).
